# Morphological study of bone marrow to assess the effects of lead acetate on haemopoiesis and aplasia and the ameliorating role of *Carica papaya* extract

**DOI:** 10.3892/etm.2012.851

**Published:** 2012-12-05

**Authors:** CHING S. THAM, SRIKUMAR CHAKRAVARTHI, NAGARAJA HALEAGRAHARA, RANJIT DE ALWIS

**Affiliations:** 1 Faculty of Medicine, University of Queensland, Brisbane, Australia;; 2 Department of Pathology, Faculty of Medicine, International Medical University, Kuala Lumpur, Malaysia;; 3 Faculty of Physiology, James Cook University, Townsville, Australia;; 4 Department of Community Medicine, Faculty of Medicine, International Medical University, Kuala Lumpur, Malaysia

**Keywords:** lead acetate, *Carica papaya*, bone marrow, haemopoiesis, aplasia

## Abstract

Lead causes damage to the body by inducing oxidative stress. The sites of damage include the bone marrow, where marrow hypoplasia and osteosclerosis may be observed. Leaves of *Carica papaya,* which have antioxidant and haemopoietic properties, were tested against the effect of lead acetate in experimental rats. The rats were divided into 8 groups; control, lead acetate only, *Carica papaya* (50 mg and 200 mg), post-treatment with *Carica papaya* (50 mg and 200 mg) following lead acetate administration and pre-treatment with *Carica papaya* (50 mg and 200 mg) followed by lead acetate administration. The substances were administered for 14 days. The effects were evaluated by measuring protein carbonyl content (PCC) and glutathione content (GC) in the bone marrow. Histological changes in the bone marrow were also observed. The results showed that *Carica papaya* induced a significant reduction in the PCC activity and significantly increased the GC in the bone marrow. *Carica papaya* also improved the histology of the bone marrow compared with that of the lead acetate-treated group. In summary, *Carica papaya* was effective against the oxidative damage caused by lead acetate in the bone marrow and had a stimulatory effect on haemopoiesis.

## Introduction

Lead acetate [(Pb(CH_3_COO)_2_] is a toxic white crystalline chemical compound made by treating lead(II) oxide with acetic acid. Lead acetate is used in the cosmetics industry as a ingredient of hair dyeing products. Lead is known to damage numerous organs in the human body, including the brain, liver and kidneys (1–3). One of the most common sources of exposure to lead is occupational exposure via inhalation and ingestion (4). Workers may inhale lead dust when sanding lead-coated surfaces or reclaiming lead from scrap metals. Certain workers also ingest lead accidentally during work when basic hygiene measures are not adhered to (4). Contaminated water and food are other routes of exposure to lead. Lead may be inhaled, ingested or absorbed through the skin. Lead has been shown to cause damage to bones, as well as the suppression of haemopoiesis. One study has shown that lead acetate causes thickening of bone, irregular bony spicules and mild osteoporotic changes (5). The same study also detected hypoplasia of bone marrow cells (5). Lead has been shown to cause damage by inducing oxidative stress. A study demonstrated that lead causes a high protein carbonyl content (PCC) which is indicative of oxidative damage and low antioxidant levels (6). Lead inhibits osteoclastic bone resorption and osteoblastic bone formation and may lead to early osteoporosis (7). Lead causes multiple types of damage to the bone and bone marrow and therefore it is important to identify a method of ameliorating this toxicity.

*Carica papaya*, the sole species in the genus *Carica* and family *Caricaceae*, is a fruit tree common to tropical countries. The fruits are sweet and juicy and the tree is hollow with large palmate leaves (8). *Carica papaya* leaves have been shown to have anti-inflammatory (9), antitumour and immunomodulatory effects (10) and antioxidant properties (11). *Carica papaya* leaves have been used traditionally to treat dengue as certain individuals who consume it exhibit healthy increases in platelet counts. Two studies have supported these findings. The first identified an increase in the thrombocyte count in mice following treatment with a papaya leaf suspension (12) and suggested that the extract helped the bone marrow produce more platelets. Another study on a dengue patient detected an increase in white blood cell and platelet counts following treatment with *Carica papaya* leaf extract (13). However, no studies have been performed to assess the stimulatory effect of *Carica papaya* in the bone marrow directly or to assess the haemopoiesis of cells. In the present study, with regard to the antioxidant properties and stimulatory haemopoietic effects of *Carica papaya*, the effects of lead acetate and *Carica papaya* in the bone marrow and haemopoiesis were investigated.

## Materials and methods

### Lead acetate preparation

Lead acetate was obtained from SYSTERM, ChemAR, Essen Haus Sdn Bhd (Selangor, Malaysia). A solution was prepared by dissolving 1 g lead acetate in 10 ml distilled water. The 100 mg/ml lead acetate solution was administered to rats at 50 mg/kg body weight. A fresh lead acetate solution was prepared every 2–3 days.

### Carica papaya extract preparation

*Carica papaya* leaves (4 kg) were collected from nurseries in Sungai Buloh, Malaysia. The leaves were washed thoroughly and dried in a pre-heated oven at 40°C for 1 day. The dried leaves were thoroughly blended into powdered form and stored in a bottle. Powdered dried papaya leaves (50 g) were weighed using an analytical balance (B204-S, S/N 1119512901; Mettler Toledo, Schwerzenbach, Switzerland) and transferred to a thimble. By using a soxhlet extractor and 95% ethanol, the ethanolic extract of the *Carica papaya* leaves was obtained. The ethanolic extract was filtered using filter paper and a conventional glass funnel. The ethanolic extract was further concentrated using a rotary evaporator (R-200, S/N 10328564; Buchi Corporation, New Castle, DE, USA) to remove the ethanol until a dark green viscous solution was observed. The concentrated green solution was stored overnight at −80°C. The solution was then freeze-dried using a freeze dryer (FreeZone 4.5, S/N 040622016S; Labconco, Kansas City, MO, USA) for 2 days. The obtained *Carica papaya* extract, which was a dark green powder, was weighed using the same analytical balance. A total of 50 g of dried papaya leaves yielded ∼10 g of *Carica papaya* extract. The *Carica papaya* extract was then stored at −80°C until used. To prepare the *Carica papaya* (50 mg) solution, 1 g *Carica papaya* extract was dissolved in 10 ml distilled water. The 100 mg/ml *Carica papaya* solution was administered to the rats at 50 mg/kg body weight. To prepare the *Carica papaya* (200 mg) solution, 4 g *Carica papaya* extract was dissolved in 10 ml distilled water. The 400 mg/ml *Carica papaya* solution was administered to rats at 200 mg/kg body weight. The powdered extract did not dissolve fully and created a dark green suspension. The mixture was agitated thoroughly before it was force-fed to the rats. The solutions were freshly prepared and used within 2–3 days.

### Experimental animals and experimental design

A total of 48 male albino rats (Sprague-Dawley strain) with body weights of 160–180 g and between 6 and 8 weeks old were obtained from the Animal Housing Facility (AHF) at The University of Malaysia. The rats were divided into eight groups (6 rats each) and each group (Table I) was housed in metal cages located in the AHF at the International Medical University. The rats were kept under normal laboratory conditions and acclimated for one week. The rats were given free access to water and fed with standard rat chow. The weights of the rats were recorded daily. All experimental procedures were in accordance with ethical guidelines for animal experimentation and the study protocol was approved by the Institute Research and Ethics Committee.

At the end of the treatment period, the rats were sacrificed. The femurs were removed and one femur was preserved in 10% neutral buffered formalin. The contents of the other femur were flushed out using a 5 ml PBS solution. The homogenised contents of the femur were then centrifuged and the supernatant was stored at −80°C until the assays were performed.

The assays that were performed on the homogenised contents of the femur were the glutathione content (GC) and PCC assays.

For the histopathological analysis, the femur in the formalin solution was decalcified using a 10% EDTA solution for 2 weeks. The femur was then processed and sectioned into 5-μm sections using a microtome. The tissue that was made into slides was stained with haematoxylin and eosin (H&E) and a reticulum stain and observed under a microscope.

### Statistical analysis

All the biochemical findings were expressed as the mean with standard deviation. The confidence interval was set at 95% with a level of significance, α, set at 0.05. The P-value was compared with α. P<0.05 was considered to indicate a statistically significant difference. All groups were compared using the Kruskal-Wallis non-parametric test. Individual groups were compared using Mann-Whitney U tests for pair-wise comparisons. The groups were compared with regard to the antioxidant levels and the PCC of the bone marrow. This enabled the effects of *Carica papaya* and lead acetate on the bone marrow to be compared.

For the histopathological results, the findings were compared in each group. The findings were confirmed by two pathologists, CST and SC. Images were obtained as evidence. The results concerning the observation of haemopoietic cells, fat cells, extent of fibrosis, sclerosis and reticulum stain fibers were also summarised in the form of a table. The observations were compared and given a score of 0, +, ++ or +++, in increasing order of severity.

## Results

### PCC assay

Using pair-wise comparison, no significant differences were observed among the control, *Carica papaya* (50 mg) and *Carica papaya* (200 mg) groups. A significant increase in PCC was observed in the lead-treated animals (P<0.05). The rats treated by lead followed by *Carica papaya* (post-treatment groups) exhibited PCCs that were significantly higher than that of the control (P<0.05) but significantly lower than that of the rats treated with lead alone (P<0.05). The rats treated with *Carica papaya* followed by lead (pre-treatment groups) exhibited PCCs that were significantly higher than that of the control (P<0.05) but significantly lower than that of the lead alone group (P<0.05). The decrease in the PCC was more pronounced in the rats treated with the higher dose of *Carica papaya* (200 mg/kg).

### GC assay

Using pair-wise comparison, no significant differences were observed among the control, *Carica papaya* (50 mg) and *Carica papaya* (200 mg) groups. A significant decrease in GC was observed in the lead-treated animals (P<0.05). The lead followed by *Carica papaya* (post-treatment) groups exhibited GCs that were significantly lower than that of the control (P<0.05) but significantly higher than that of the lead alone group (P<0.05). The *Carica papaya* followed by lead (pre-treatment) groups exhibited significantly higher GCs than the lead alone group (P<0.05). The increase in GC was more pronounced in the groups treated with the higher dose of *Carica papaya* (200 mg/kg).

### Histopathological analysis

**Figure 1.**
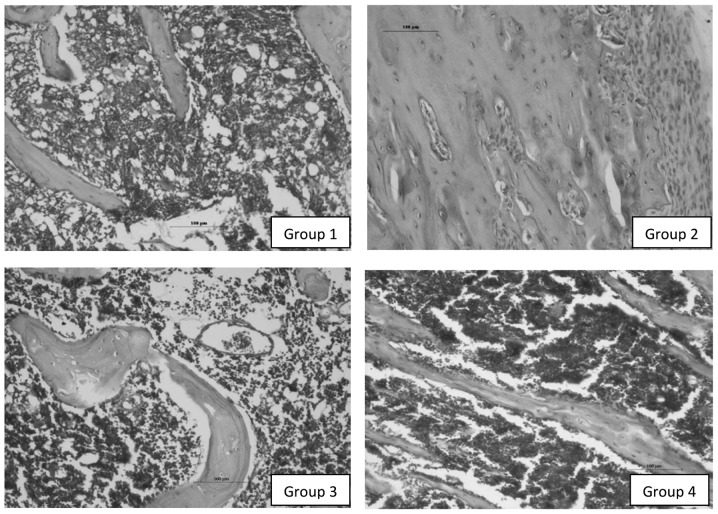
Photomicrographs of tissue sections (×200 magnification, H&E stained). (Group 1) Healthy cortical bone, haemopoietic cells and fat cells. (Group 2) Areas of sclerosis and areas of fibrosis. (Group 3) Prominent blood vessel and areas of mild hyperplasia of haemopoietic cells. (Group 4) Hyperplasia of haemopoietic cells surrounded by healthy cortical bone. Group 1, control; group 2, lead alone; group 3, *Carica papaya* alone (50 mg/kg); group 4, *Carica papaya* alone (200 mg/kg); H&E, haematoxylin and eosin.

**Figure 2.**
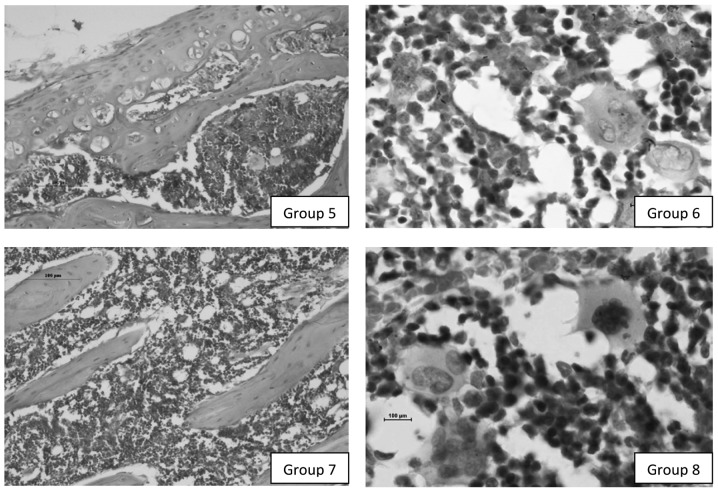
Photomicrograph of tissue sections (H&E stained). (Group 5) Proliferation of cells beginning from the metaphysis of the bone marrow and hyperplasia of the haemopoietic cells (×200 magnification). (Group 6) Increase in megakaryocytes and myeloblast cells. Erythroid precursors may also be observed (×1000 magnification). (Group 7) Increased blood vessel proliferation, hyperplasia of the cells and a healthy cortical bone (x200 magnification). (Group 8) Increase in megakaryocytes and eosinophils as well as myeloblast precursors. Reticulocytes may also be observed (×1000 magnification). Group 5, lead and post-treatment with *Carica papaya* (50 mg/kg); Group 6, lead and post-treatment with *Carica papaya* (200 mg/kg); Group 7, pre-treatment with *Carica papaya* (50 mg/kg) followed by lead; Group 8, pre-treatment with *Carica papaya* (200 mg/kg) followed by lead; H&E, haematoxylin and eosin.

The controls showed healthy cortical bones with normal and adequate bony spicules. They also exhibited satisfactory haemopoiesis and no areas of fibrosis were observed. There were also normal amounts of reticulin fibres. In the lead acetate alone group, there was strong evidence of fibrosis and focal areas of sclerosis. Significant marrow hypoplasia was also detected. There were coarse reticulin fibres with minimal cells (Fig. 1). In the *Carica papaya* alone groups, there were healthy cortical bones with normal architecture, focal areas of mild hyperplasia and normal amounts of reticulin fibres. One of the distinct features identified was the mild increase in the levels of eosinophils and their precursors in the bone marrow. The lead exposure followed by *Carica papaya* groups showed abundant osteoid formation, along with hyperplasia of the marrow, pushing out the cortical bone (Fig. 2). The cortical bone was healthy and rimmed by active osteoblasts. There were also areas of fibrosis and sclerosis and an increased amount of reticulin fibres. The *Carica papaya* followed by lead exposure groups showed evidence of bone marrow morphological changes which were suggestive of lead-induced damage with alternating areas of regenerative change. There were areas of alternative marrow hypoplasia and focal islands of hyperplasia of the haemopoietic components. Mild osteosclerosis and focal areas of fibrosis were observed as well as an increased amount of reticulin fibres (Table II).

## Discussion

Lead is known to induce damage via oxidative stress. This means that lead increases the reactive oxygen species content of the body and depletes antioxidant enzyme levels (14–16). PCC is a useful biomarker indicative of oxidative damage (17,18). PCC are generated by the actions of reactive oxygen species. Hence, an increase in PCC is directly associated with increased reactive oxygen species. Glutathione is an important antioxidant in the body. A low level of glutathione indicates depletion of antioxidants while a higher level of glutathione signifies higher antioxidant levels (18).

In the biochemical results, the control and *Carica papaya* alone groups exhibited low PCC and high GC, indicating a low amount of oxidative damage with high antioxidant levels. High PCC and low GC were observed in the lead acetate alone group, indicating heavy oxidative damage and low antioxidant levels. This observation regarding lead acetate is consistent with previous studies which noted that bone marrow PCC was significantly higher following treatment with lead acetate and antioxidant levels were significantly lower (5,6).

In a previous study, *Carica papaya* leaves were reported to offer protection against alcohol-induced oxidatative damage to the gastric mucosa (19). In the present study, the aim was to observe whether *Carica papaya* had the ability to ameliorate oxidative damage induced by lead acetate in the bone marrow. *Carica papaya* demonstrated protective effects on lead acetate-induced oxidative damage in the bone marrow since the pre-treatment (*Carica papaya* followed by lead acetate) groups exhibited significantly lower PCC and significantly higher GC than the lead acetate alone group (P<0.05).

*Carica papaya* was also observed to have a reversal effect on lead acetate-induced oxidative damage in the bone marrow since the post-treatment (lead acetate followed by *Carica papaya*) group showed significantly lower PCC and significantly higher GC than the lead acetate alone group (P<0.05). This is consistent with a study that detected the antioxidant properties of *Carica papaya* leaves (11). Statistical analysis showing a significant difference further supported the results.

Histopathological analysis was performed to study the morphological damage and reversal of any changes in the experimental groups. It was observed that in the lead alone group there was extensive damage to the bone and bone marrow, with fibrosis, sclerosis, hypoplasia and loss of marrow space. This observation was similar to that of a previous study where the lead acetate-induced group exhibited thickening of bone, irregular bony spicules and mild osteoporotic changes (5).

The *Carica papaya* alone groups were very similar to the control group and did not show any morphological changes in terms of damage. The higher dose of *Carica papaya* exhibited improved cellularity but this was statistically insignificant compared with the lower dose (*Carica papaya* 50 mg); both doses showed adequate cellularity and healthy marrow. There was also mild hyperplasia in the 2 groups which was more prominent for the 200 mg dose. This suggests that *Carica papaya* alone has a stimulatory effect on the haemopoiesis of cells in the bone marrow.

The post-treatment (lead acetate followed by *Carica papaya*) groups exhibited areas of damage induced by lead mixed with regenerative areas having good marrow cellularity. There were zones of fibrosis intermingled with zones of hyperplastic marrow tissue. This suggests that *Carica papaya* has a reversal effect on the lead acetate-induced effects in the bone marrow, as well as a stimulatory effect on the haemopoiesis of cells in the bone marrow.

The pre-treatment (*Carica papaya* followed by lead acetate) groups also showed good cellularity with focal areas of damage observed as mild to moderate fibrosis. These groups showed areas of marrow hypoplasia intermingled with areas of hyperplasia of the bone marrow. This suggests that *Carica papaya* has a protective effect on the lead acetate-induced effects in the bone marrow and also a stimulatory effect on the haemopoiesis of cells in the bone marrow. The findings for each group were supported by reticulum staining.

Of note was the presence of zones of hyperplasia in the *Carica papaya* alone, pre-treatment and post-treatment groups which suggested that *Carica papaya* had a stimulatory effect on the haemopoiesis of cells.

Also observed was an increase in the levels of all blood cell precursors, particularly the megakaryocytes and the myeloblast series, in all pre treatment and post treatment groups (groups 5, 6, 7 and 8). Certain areas contained giant megakaryocytes and there was an increase in eosinophils. Increased proliferation of blood vessels was also observed which may have contributed to the hyperplasia of the bone marrow cells. The study was a double-blind experiment whereby the slides were selected at random and observed by two researchers.

Studies concerning the effect of *Carica papaya* on the lead acetate-induced changes to bone marrow are lacking. However, a study by Sathasivam *et al* demonstrated an increase in thrombocyte count of mice following the administration of a papaya leaf suspension which may be consistent with the present observation of an increase in the megakaryocyte count which may lead to an increased thrombocyte count (12). This observation also agrees with the finding of Sathasivam *et al* that platelet counts 72 h after dosing were significantly higher, indicating an increase in white blood cells and platelets which normalise clotting and repair the liver (12). This is consistent with the present findings of a notable increase in white blood cells precursors (myeloblast series) and increase in the megakaryocyte count for platelet production in all groups treated with *Carica papaya*.

A study on a dengue patient revealed increased white blood cell and platelet counts following treatment with *Carica papaya* leaf extract (13). This also agrees with the present findings of increased levels of white blood cell precursors (myeloblast series) and megakaryocytes in all groups treated with *Carica papaya*. These findings suggest that *Carica papaya* may protect against and reverse the lead acetate-induced effects on the bone marrow, such as marrow hypoplasia, and stimulate haemopoiesis of the cells, particularly the myeloblasts and megarkaryocytes.

## Figures and Tables

**Table I.** Experimental groups.

Group	Substance	Dose (mg/kg body weight/day^a^)	Duration (days)
1	Distilled water	1	14
2	Lead acetate	50	14
3	*Carica papaya* extract	50	14
4	*Carica papaya* extract	200	14
5	Lead acetate + *Carica papaya*	50+50	14+14
6	Lead acetate + *Carica papaya*	50+200	14+14
7	*Carica papaya* + lead acetate	50+50	14+14
8	*Carica papaya* + lead acetate	200+50	14+14

a With the exception of group 1 which is presented in ml/day.

**Table II.** Morphological changes in rat femur sections.

Group	BM cells	Fat cells	Fibrosis	Sclerosis	Reticulin fibers
1	++	+	0	0	0
2	+	++	+++	+++	+++
3	+++	+	0	0	0
4	+++	+	0	0	0
5	+++	+	++	+	++
6	+++	+	+	+	++
7	+++	+	++	+	++
8	+++	+	+	+	++

Changes are scored 0, +, ++ and +++, in increasing order of severity. Group 1, control; Group 2, lead alone; Group 3, *Carica papaya* alone (50 mg/kg); Group 4, *Carica papaya* alone (200 mg/kg); Group 5, lead and post-treatment with *Carica papaya* (50mg/kg); Group 6, lead and post-treatment with *Carica papaya* (200 mg/kg); Group 7, pre-treatment with *Carica papaya* (50 mg/kg) followed by lead; Group 8, pre-treatment with *Carica papaya* (200 mg/kg) followed by lead; BM, bone marrow.
